# Effects of Annealing Conditions on the Catalytic Performance of Anodized Tin Oxide for Electrochemical Carbon Dioxide Reduction

**DOI:** 10.3390/nano15020121

**Published:** 2025-01-16

**Authors:** Nicolò B. D. Monti, Juqin Zeng, Micaela Castellino, Samuele Porro, Mitra Bagheri, Candido F. Pirri, Angelica Chiodoni, Katarzyna Bejtka

**Affiliations:** 1 Center for Sustainable Future Technologies @POLITO, Istituto Italiano di Tecnologia, Via Livorno 60, 10144 Turin, Italy; 2 Department of Applied Science and Technology, Politecnico di Torino, C.so Duca degli Abruzzi 24, 10129 Turin, Italy

**Keywords:** electrochemical CO_2_ reduction, SnO_2_ catalyst, mesoporous, HCOOH production, oxygen vacancy, annealing

## Abstract

The electrochemical reduction of CO_2_ (CO_2_RR) to value-added products has garnered significant interest as a sustainable solution to mitigate CO_2_ emissions and harness renewable energy sources. Among CO_2_RR products, formic acid/formate (HCOOH/HCOO^−^) is particularly attractive due to its industrial relevance, high energy density, and potential candidate as a liquid hydrogen carrier. This study investigates the influence of the initial oxidation state of tin on CO_2_RR performance using nanostructured SnO_x_ catalysts. A simple, quick, scalable, and cost-effective synthesis strategy was employed to fabricate SnO_x_ catalysts with controlled oxidation states while maintaining consistent morphology and particle size. The catalysts were characterized using SEM, TEM, XRD, Raman, and XPS to correlate structure and surface properties with catalytic performance. Electrochemical measurements revealed that SnO_x_ catalysts annealed in air at 525 °C exhibited the highest formate selectivity and current density, attributed to the optimized oxidation state and the presence of oxygen vacancies. Flow cell tests further demonstrated enhanced performance under practical conditions, achieving stable formate production with high faradaic efficiency over prolonged operation. These findings highlight the critical role of tin oxidation states and surface defects in tuning CO_2_RR performance, offering valuable insights for the design of efficient catalysts for CO_2_ electroreduction to formate.

## 1. Introduction

Electrosynthesis of value-added chemicals and fuels using CO_2_ as a raw material has attracted tremendous interest, since it allows mitigating the accumulation of CO_2_ in the atmosphere and simultaneously favoring the implementation of renewable electricity [1]. Despite intensive efforts in academic research, the industrialization of electrochemical CO_2_ reduction reaction (CO_2_RR) still encounters many challenges related to high energy barriers, multiple parallel reactions, and competitive hydrogen evolution reaction (HER) [2]. Consequently, CO_2_RR products include a wide range of chemicals, such as carbon monoxide (CO), formate (HCOO^−^), methane (CH_4_), methanol (CH_3_OH), ethane (C_2_H_6_), ethylene (C_2_H_4_), and ethanol (C_2_H_5_OH) [3,4,5,6,7,8,9,10,11,12,13,14]. Among them, formic acid and formate (HCOOH and HCOO^−^) are considered attractive, since they are widely used in many industries, such as leather, rubber, medicine, and fibre. In addition, they are promising liquid hydrogen energy carriers due to the high energy density of 1.77 kWh L^−1^, comparable to the commercial high pressure hydrogen tanks [15]. They are also used as fuel in direct formate fuel cells [16]. Most particularly, the short-chain simple building-block molecule formate is currently the most compelling CO_2_RR product beyond CO from a techno-economic analysis [17]. Hence, formate is one of the most desired products from the electrochemical reduction of CO_2_.

The acquisition of target products of CO_2_RR calls for appropriate catalysts able to selectively drive the reaction, and great efforts have been dedicated to the development of electrocatalysts for formate production [18,19]. From the pioneer work of Hori et al. [20], some metals, including lead (Pb), mercury (Hg), indium (In), bismuth (Bi) and tin (Sn), are selective for HCOO^−^ formation. Among them, Sn and SnO_x_ have become the most interesting and benchmark catalysts for this purpose due to their high selectivity, good activity, non-noble, eco-friendly, and low-cost characteristics [21]. The intensive studies show that the performance of Sn-based materials is highly tunable through morphology and nanostructure engineering, doping, alloying and defects creation [6,22,23]. In our previous work, we reported a mesoporous SnO_2_ nanocrystals with abundant grain boundaries, high specific surface area, and easily accessible porosity, resulting in being active and selective for the CO_2_RR [24]. By doping with titanium (Ti) and zinc (Zn), the activity of the SnO_2_ toward CO_2_RR can be enhanced [6]. Particularly, the oxidation state of tin, which could be modified by, for example, fine-tuning the annealing conditions, is proven to play an essential role in the selectivity and activity of the electro conversion of CO_2_ to formate [25,26], and both experimental and theoretical calculations suggested that the SnO_x_ layer is the catalytic active site [27,28,29,30]. The studies with operando techniques demonstrated that metastable hydrous Sn oxides exist on the surface of Sn electrode, despite the operating potentials for CO_2_ reduction being thermodynamically unstable for Sn oxides [27,28,31,32].

In order to study the influence of the initial oxidation state of tin on the CO_2_RR, the present work develops a facile and low-cost strategy to fabricate different nanostructured SnO_x_ catalysts with altered oxidation states, while keeping the morphology and particle size unchanged. The composition, morphology, and crystal structure of the SnO_x_ nano-catalysts were characterized using scanning electron microscopy (SEM), transmission electron microscopy (TEM), X-ray diffraction (XRD), Raman, and X-ray photoelectron spectroscopy (XPS). Chronoamperometric measurements with product analysis were conducted to elucidate the catalytic performance of various materials for CO_2_RR. This work represents an important advancement toward the understanding of the composition–performance relationship of Sn catalysts for efficiently converting CO_2_ to formate. It highlights how variations in oxidation state, structure, and the effect of oxygen vacancy influence the activity and selectivity of the CO_2_RR. The optimal catalyst, with higher oxygen vacancy content, reported selectivity towards CO_2_RR products > 90% with a current density > 60 mA cm^2^ for more than 30 h.

## 2. Materials and Methods

### 2.1. Synthesis of the Catalysts

The catalyst was synthesized via anodic oxidation of 0.5 mm thick tin foil (99.95% purity, Advent RM, Oxford, UK) followed a by peeling-off step. Tin foil was cut into 2.5 × 2 cm^2^ pieces, cleaned via sonication in acetone, and then in ethanol, and subsequently dried. Oxidation was performed at 10 V in a 0.3 M NaOH solution (≥98.0 wt%, Sigma–Aldrich, Saint Louis, MO, USA) for 30 min, using a platinum foil as the cathode. The resulting oxide layer was peeled off through sonication, followed by the annealing of the powder samples at temperatures of 370 °C and 525 °C for 2 h under atmospheric and nitrogen conditions. Thermal treatment was performed in a Nabertherm LT 15/12/P330 muffle furnace (Nabertherm, Lilienthal, Germany) in air or in a Horizontal Quartz Tube Furnace (Carbolite Gero Ltd., Derbyshire, UK) under a controlled nitrogen atmosphere. The samples were labelled according to their annealing parameters as follows: pristine, 370 °C, 525 °C, 525 °C_N2_.

### 2.2. Physical and Chemical Characterizations of the Catalysts

Field Emission Scanning Electron Microscopy (FESEM, ZEISS Auriga, Oberkochen, Germany) was used to evaluate the morphology of the as-grown material and prepared electrodes. Samples for Transmission Electron Microscopy (TEM) were prepared by drop-coating a solution of obtained material dispersed in ethanol onto a holey carbon-coated Cu grid. TEM observations were performed with an FEI Tecnai F20ST microscope (Thermo Fisher Scientific, Waltham, MA, USA), equipped with a field emission gun (FEG) operated at 200 kV.

X-ray diffraction (XRD) was performed in Bragg–Brentano symmetric geometry using a PANalytical X’Pert Pro instrument (Malvern Panalytical, Malvern, UK) (Cu-Kα radiation, 40 kV and 30 mA) equipped with an X’Celerator detector. The catalyst powder was deposited on a glass slide to avoid signal contributions from carbonaceous substrates or other extraneous components. The XRD patterns were analyzed by means of Rietveld refinement with the use of the MAUD software (version 2.94) [33,34]. The diffractometer instrumental function was determined by analyzing the XRD pattern of the LaB_6_ standard provided by the National Institute of Standards and Technology. The structural model used for SnO_2_ is Crystallography Open Database (COD) ID: 9007497, while for the Sn the reference model is COD ID: 1524713. The background shows evidence of amorphous contribution. For each sample, the crystallite size and micro-strain were assumed to be isotropic.

Raman spectroscopy was carried out with a Renishaw inVia Reflex micro-Raman (Renishaw, Gloucestershire, UK) spectrophotometer equipped with a cooled charge-coupled device camera, and excited with a 514.5 nm wavelength solid state laser source.

X-ray photoelectron spectroscopy (XPS) analysis has been performed using a PHI 5000 Versaprobe spectrometer (Physical Electronics, Chanhassen, MN, USA). A monochromatic Al k-alpha X-ray source has been used (1486.6 eV). Survey and High Resolution (HR) spectra have been acquired to obtain information related to the presence of chemical elements and, secondly, to achieve more information regarding each element’s chemical states due to the surrounding environment. Pass energies of 187 eV and 23 eV have been used for survey and HR spectra, respectively. A double ion/electron charge compensation source has been applied to reduce surface charging phenomena due to non-perfectly conductive material. The C1s peak has been positioned to 284.8 eV as a reference for the Binding Energy (BE) axis, as adventitious carbon, as reported in the literature [35]. Multipak version 9.7 dedicated software has been used to analyze raw data.

### 2.3. Preparation of the Electrodes

To prepare the SnO_x_-based gas diffusion electrode, an ink was prepared by mixing 10 mg of the catalyst, 1.5 mg of carbon black (Vulcan XC-72R, Cabot, Boston, MA, USA), 320 µL of isopropyl alcohol, and 100 µL of Nafion 117 solution (5 wt.%, Sigma-Aldrich). The resulting catalyst ink was drop-casted onto a carbon paper gas diffusion layer (SIGRACET 28BC, SGL Technologies, Meitingen, Germany) to achieve a final loading of 3 mg cm^−2^. The active area of the electrodes was 1.5 cm^2^.

### 2.4. Electrochemical Tests and Product Analysis

The electrodes were evaluated in two different configurations. Initially, a screening of the different samples was performed in a custom H-cell (Disa Raffaele e F.lli snc, Sesto San Giovanni, Milano, Italy). The resulting optimal catalyst was further tested in a flow cell (ElectroCell Europe A/S, Tarm, Denmark); these two configurations are schematised in Figure S1. In both configurations, a Nafion membrane 117 was used, and the electrolyte was an aqueous solution of KHCO_3_ (Sigma ≥ 98.0 wt%). The catalysts’ performance has been investigated by applying several potentials: −0.8 V, −1.0 V, and −1.2 V versus the Reversible Hydrogen Electrode (RHE) with a CHI760D electrochemical workstation. The potential conversion to RHE is possible thanks to Nernst’s equation: E_vs.RHE_ = E + E_Ag/AgCl (3M Cl^−^)_ + 0.0591 × pH. Unless specified, all reported potentials in this work refer to the RHE.

Catalyst performances are evaluated according to geometrical current density and faradaic efficiency towards CO_2_RR products. Using the following equations:

$$FE_{Gas}=\frac{ppm\;V_{Gas}\;n{}^{\circ}\;F}{Q\;V_{M}},$$

$$FE_{Liquid}\;=\frac{ppm\;\rho \;n{}^{\circ}\;F\;V_{Liquid}}{Q\;W_{M}},$$

where ppm is the product concentration (ppm) in volume, n° is the number of electrons requested in the reaction, and F is the Faraday’s constant (C mol^−1^). In the first equation, $V_{Gas}$ is the gas flow rate entering in the GC (L min^−1^), considered over 1 min, $V_{M}$ is the molar volume for perfect gas constant (L mol^−1^), and Q is the total charge consumed by the reduction reactions during the test in one minute (C). In the second equation, $V_{Liquid}$ is the catholyte volume (mL), ρ is the product density (g mL^−1^), and $W_{M}$ is the product molar weight (g mol^−1^). Q  is the total charge consumed by the reduction reactions during the period for the liquid product accumulated (C).

Electrochemical product quantification was performed online using a micro gas chromatograph (uGC) and high-performance liquid chromatography (HPLC). The gaseous products, including H_2_, CH_4_, CO, C_2_H_4_, and C_2_H_6_, were analyzed using an Inficon uGC Fusion system equipped with an Igeni filter, a 10 m Rt-Molsieve 5A column, and a microthermal conductivity detector. For the quantification of C_2_ hydrocarbons specifically, an 8 m Rt–Q Bond column was used.

The liquid-phase products were quantified using an HPLC system (Nexera Series, Shimadzu, Kyoto, Japan), equipped with a Photodiode Array Detector and a Refractive Index Detector. The system employed a ReproGel H+ column (300 × 8 mm, Dr. Maisch) with a precolumn. The HPLC method had a runtime of 20 min, with 10 μL of the sample injected into a mobile phase composed of 9.0 mM H_2_SO_4_, flowing at a rate of 1.0 mL min^−1^. The entire system was maintained at 60 °C.

## 3. Results

### 3.1. Physical/Chemical Properties of Different Catalysts

**Figure 1:**
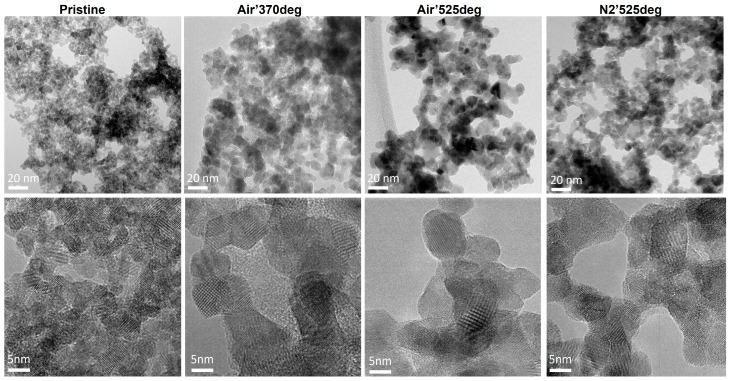
TEM characterization of all studied catalysts: top line BF-TEM and bottom line HRTEM for Pristine, Air′370 °C, Air′525 °C and N2′525 °C.

Figure 1 and Figure S2 show the morphology of the as-grown catalyst. It consists of an irregular porous structure, as observed from the top view FESEM image (Figure S2A), and by channels, which are characteristic of oxides prepared by the same growth method [36,37]. The channels are irregular and oriented perpendicular to the substrate surface, as evidenced in the cross-section view (Figure S2B). Such porous structure provides high availability of catalytic sites due to high surface area and porosity, which is of high profit to the CO_2_RR application [38,39,40].

The studied samples were obtained through calcination of the as-grown catalyst in air and in N_2_ atmosphere. The morphology at the nanoscale was characterized in detail by transmission electron microscopy (TEM), and the main results are presented in Figure 1. Bright Field TEM image shows that the pore walls of the SnO_x_ prepared via anodic oxidation consist of small NPs, which is in line with the FESEM observation.

The ring pattern in Selected Area Electron Diffraction (SAED), shown in Figure S3, analyzed with the Circular Hough analysis tool of Digital MicrographTM version 3.51.3720.0 software [41], confirms the polycrystalline nature of the sample and the presence of the SnO_2_ (tin oxide, JCPDS 00-041-1445) crystalline phase. HRTEM reveals that these NPs are chain-like connected nanocrystals, with their size increasing with calcination temperature for the samples treated in air, as expected [24]. A different behavior was observed for the sample treated in N_2_, which showed a lower NPs size increase compared to the sample treated in air. A similar behavior was observed by Jeng in SnO_2_ films prepared by sol–gel [42]. The XRD results indicate that the presence of oxygen during annealing enhances the precipitation of SnO_2_ phase, compared to annealing in inert conditions (N_2_). Variation in ambient conditions, and therefore oxygen availability, during the heating experiment can lead to different outcomes in terms of the crystal size and phase transformation, and other material properties relevant to CO_2_RR, including resistivity, as shown by the authors of [42,43].

**Figure 2:**
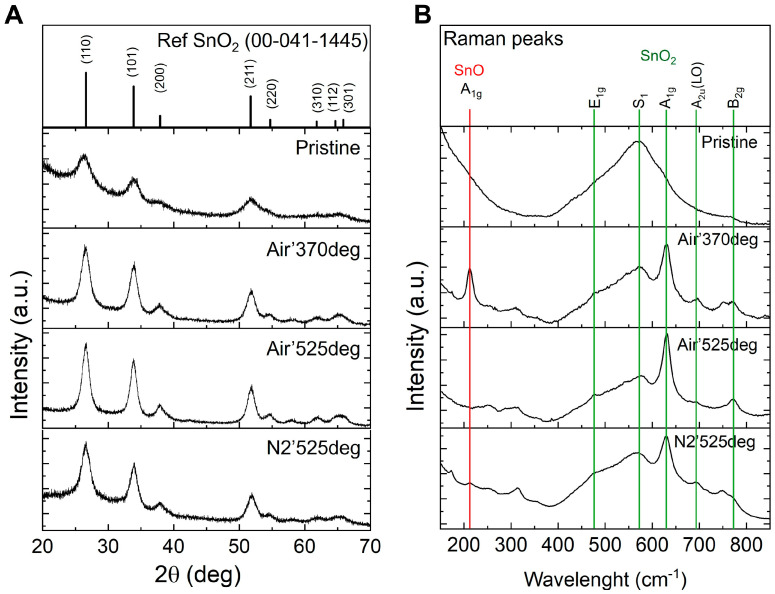
XRD patterns (**A**) and Raman spectra (**B**) obtained from each sample.

XRD analysis was performed to study the crystalline phase of the samples. The XRD patterns of the powders are shown in Figure 2A. The as-grown sample is slightly crystalline and shows an amorphous background. The peaks are rather broad, indicating their small crystalline size. The peaks of the SnO_x_ sample correspond to the reflections related to (110), (111), (200), (211), (220), and (311), and the planes of crystalline SnO_2_ (Tin Oxide, JCPDS 00-041-1445). A higher crystallization degree of SnO_x_ occurs after treatment in air at a temperature of 370 °C, with XRD peaks becoming progressively narrower as the calcination temperature increases. By increasing the annealing temperatures, the diffraction peaks appear sharpened and enhanced, thereby indicating that the particles have grown and the crystal quality has been improved.

However, a similar trend was not observed for the sample treated at 525 °C in N_2_, which shows broader XRD peaks than the samples treated in air.

To gain further insight into the crystalline structure, the XRD patterns are fitted by Rietveld refinement using the structural models reported in the experimental section, and the crystallite sizes obtained from refined parameters are shown in Table 1. Although a significant change in the lattice parameters was not observed, an increase in the average coherent crystalline domain size was observed for all samples.

Annealing temperature plays a crucial role in the formation and relaxation of strain in polycrystalline SnO_x_. XRD peak shifts reveal that lower annealing temperatures result in higher strain due to limited defect relaxation and smaller grain sizes. Using the MAUD program for strain analysis, it was observed that higher temperatures promote grain growth and defect healing, reducing strain. These findings, shown in Table 1, align with literature [44].

Therefore, we can conclude that the annealing conditions, including temperature and the oxidating atmosphere, control the morphology, nanoparticle size, crystallinity, and composition of the Sn-based catalysts.

For further investigation of the crystalline structure of SnO_x_ catalysts, Raman scattering spectroscopy was employed. Raman scattering is sensitive to the local structure and crystal surface area and is a powerful tool for the characterization of nanomaterials and a qualitative probe for the presence of lattice defects in solids. The Raman spectra show that the as-prepared SnO_x_ catalysts do not exhibit obvious Raman modes (Figure 2B), which is consistent with their principally amorphous nature.

The Raman spectrum of the catalyst annealed at 370 °C shows the presence of numerous vibrational peaks. The most intense peaks are the following: A1g mode of SnO at 212 cm^−1^, A1g, and B2g vibrational modes of SnO_2_ at 631 and 772 cm^−1^, respectively. Other, less dominant modes at 475, 560, and 694 cm^−1^ correspond to the E1g mode, S1 band, and A2u (LO) vibrational modes of SnO_2_, respectively [45,46,47,48]. These Raman bands further confirm the characteristics of the tetragonal rutile structure and the very small crystal size of the SnO_2_. The intensities of the SnO_2_-related peaks had significantly increased when the annealing temperature was increased to 525 °C, while those related to SnO are not present anymore.

The catalyst annealed at 525 °C in an inert atmosphere exhibits the above-mentioned modes, with the 212 cm^−1^ mode of SnO showing very low intensity. The SnO_2_ modes differ in relative intensity with respect to the sample annealed in air at the same temperature. For instance, the S1 band, related to disorder, is more intense. Also, additional small peaks were observed at around 170 and 315 cm^−1^, which, according to the literature, can be assigned to Sn_3_O_4_ (with Raman-active modes at 90, 140, 170, and 240 cm^−1^ [49,50]) and Sn_2_O_3_ (with vibrational frequencies at 76, 122, 235, and 300 cm^−1^ [50]). The Sn_3_O_4_ and Sn_2_O_3_ intermediate oxides have triclinic structures that are theoretically formed when some layers of oxygen are removed from the (101) planes of rutile SnO_2_ [51,52].

The analysis of Raman spectra confirmed that the crystallization of SnO_x_ can be improved by annealing the as-grown material, and that annealing in oxidizing condition (the presence of air) leads to the crystallization of pure tetragonal rutile SnO_2_ structures, while annealing in inert atmosphere leads to the crystallization of mixed Sn oxide structures. In addition, Raman analysis confirmed that crystallization is more efficient at higher temperatures.

**Figure 3:**
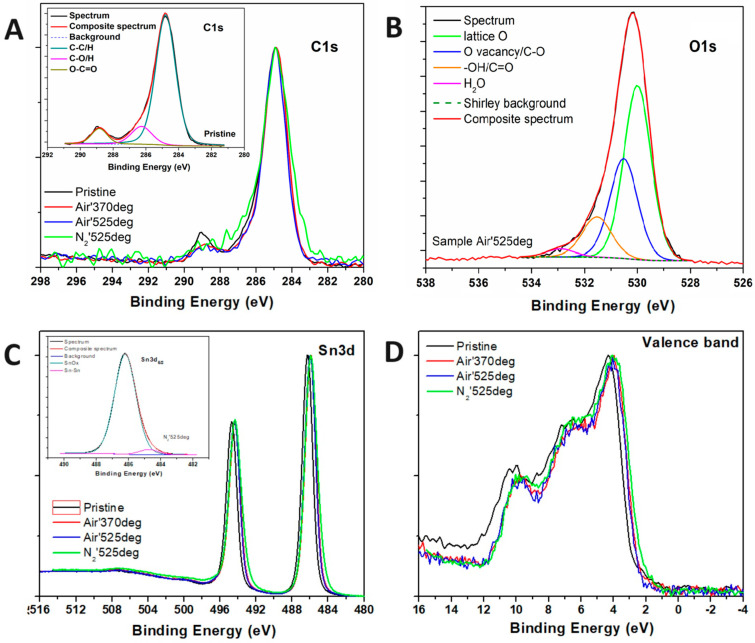
XPS HR spectra for (**A**) C1s, (**B**) O1s, (**C**) Sn3d, and (**D**) Valence Band regions.

To obtain more information related to the surface composition of these catalysts, XPS measurements were performed. A first survey scan has been acquired for each of the four materials produced (not reported), which highlighted the presence of C, O and Sn, and the absence of any other chemical species. Carbon has been assigned to environmental contamination, thus using its position as a reference by putting it at 284.8 eV, as adventitious C. Relative atomic concentrations have been calculated and reported in Table 2. It can be observed that, starting from the pristine material, the annealing process reduces the level of surface contamination by decreasing the relative amount of C. In particular, the sample annealed at 525 °C in N_2_ shows the lowest amount of C (10.4 at.%). Since adventitious C usually presents chemical shifts due to C–O bonds, HR spectra have been acquired for both C1s and O1s regions (see Figure 3). By increasing the annealing temperature, a reduction in the Carbon–Oxygen bonds can be seen, especially in the O–C=O bond region above 288 eV. The fitting procedure applied to C1s regions (see Figure 3A inset for an example) allowed us to calculate the percentage of O involved in bonds with C and the remaining portion due to oxides [53]. Accordingly, in Table 2, the Oxygen column is separated into two sub-columns, one related to C–O bonds and the other related to oxides. As can be inferred from Table 2, most of the O1s peak corresponds to the chemical shift of the oxides. The O1s peaks show four different regions, according to Dai et al. and other authors: one related to the O atom in the oxide lattice, a second due to O vacancy together with C–O bonds, a third one due to adsorbed species, i.e., -OH, together with -C=O, and the latter due to water residues [54,55]. In Figure 3B, we have reported that the O1s region related to the sample annealed in air at 525 °C, together with the deconvoluted peaks, in agreement with the chemical shifts just introduced. The other three O1s regions, for the remaining samples, are reported in Figure S4. By comparing the intensity of the O vacancy component, for all the four samples, we noticed that the sample annealed at 525 °C in air is the one with the highest intensity (31.2%), while the sample annealed at 525 °C in inert atmosphere is the one with the lowest intensity (27.4%). We have summarized the O1s fitting procedure results in Table S1. After also having acquired the Sn3d doublet region, we could evaluate also the O_oxide_/Sn_oxide_ ratio, which is reported in the last column of Table 2. The pristine sample shows a ratio of 2.08, while the ratio progressively decreases by increasing the annealing temperature in air, down to a value of 1.93 for the sample annealed in air at 525 °C. The sample annealed in inert atmosphere at 525 °C shows an increase in the ratio up to 2.08, compared with the sample annealed in air at the same temperature. In conclusion, annealing in air at high temperature contributes to a deficiency of O abundance at the surface if referred to the 1:2 expected ratio for SnO_2_.

It must be underlined that, due to the overlapping chemical shifts of SnO_2_ and SnO compounds, the XPS analysis of the Sn3d doublet region is particularly difficult, leading to a problematic segregation of these two species. According to the literature, several chemical shifts can be reported for these two species: Sn3d_5/2_ position for SnO is in the range of (486.5 ± 0.6) eV, while for SnO_2,_ it is located at (486.7 ± 0.3) eV. The material analyzed in the present work shows corresponding peaks in the (485.9–486.2 eV) range. Luckily, the Sn-Sn chemical shift is positioned at lower binding energy values, (485.0 ± 0.5) eV, and can be clearly distinguished from the oxidized states [56]. In fact, while pristine and air-calcined samples show a single component for each peak of the Sn3d doublet (see Figure 3C), with FWHM values in the 1.36–1.45 eV range, the sample annealed in an inert atmosphere shows an asymmetric peak, with FWHM = 1.64 eV, thus implying the presence of more than one single component to completely overlap the raw data with the composite spectrum. A second component (see inset in Figure 3C) has been added at 484.7 eV (5.3% of the total amount of Sn3d signal) and can be assigned to Sn-Sn chemical shift, as reported by R. Shiratsuchi et al. [57]. This last finding is in accordance with other measurements performed on the sample annealed in the inert atmosphere, which showed the presence of mixed phases. To define the oxide species of tin oxide, Figure 3D reports the analysis of the Valence Band (VB) region, in which the highest occupied states near the Fermi Energy level (BE = 0 eV) show different curve shapes according to SnO_2_ or SnO [58]. All samples analyzed in the present work show the typical band structure assigned to SnO_2_, with a small shift (Δ = 0.3 eV) towards lower BE values for the annealed samples, which means that the distance between the Valence Band Maximum (VBM) and the Fermi level has been reduced.

The dynamic nature of SnO_x_ catalysts during CO_2_RR is a critical consideration, as their performance is influenced not only by their initial state but also by transformations that occur under reaction conditions. Numerous in situ characterization studies have demonstrated the ability to analyze catalysts in real time, revealing active sites and reaction mechanisms by detecting intermediates under reactive conditions. Techniques such as Raman spectroscopy [27,28,59], ATR-IR spectroscopy [31], and X-ray absorption spectroscopy [27,60] show that at potentials lower than 0.79 V vs. RHE, SnOx catalyst particles may transition from their initial Sn(IV) state to Sn(II) or, in extreme cases, to metallic Sn. This transformation is often accompanied by a decrease in Faradaic efficiency (FE) for formate production. On metallic Sn, the competing hydrogen evolution reaction (HER) dominates, making CO_2_RR less favorable. These findings indicate that CO_2_RR to HCOOH and CO occurs on metastable surfaces, with multiple reaction pathways identified for tin oxide, as discussed later in this article [61,62,63]. In our previous studies, we examined morphological and structural changes after a 20 min reduction treatment of the SnO_x_ catalysts [24]. FESEM revealed no significant morphological differences post-treatment, while XRD showed small peaks corresponding to the metallic Sn crystalline phase. TEM confirmed the absence of morphological changes. These observations suggest that catalysts prepared via anodic oxidation undergo gradual transformation during CO_2_RR.

### 3.2. Electrochemical Measurements and Product Analysis

The catalysts’ electrochemical performance toward CO_2_RR was evaluated through chronoamperometry at (−0.8 V, −1.0 V, −1.2 V vs. RHE), carried out in CO_2_-saturated electrolyte 0.1 M KHCO_3_. Catalyst performances were investigated on three independent electrodes to ensure reproducibility.

**Figure 4:**
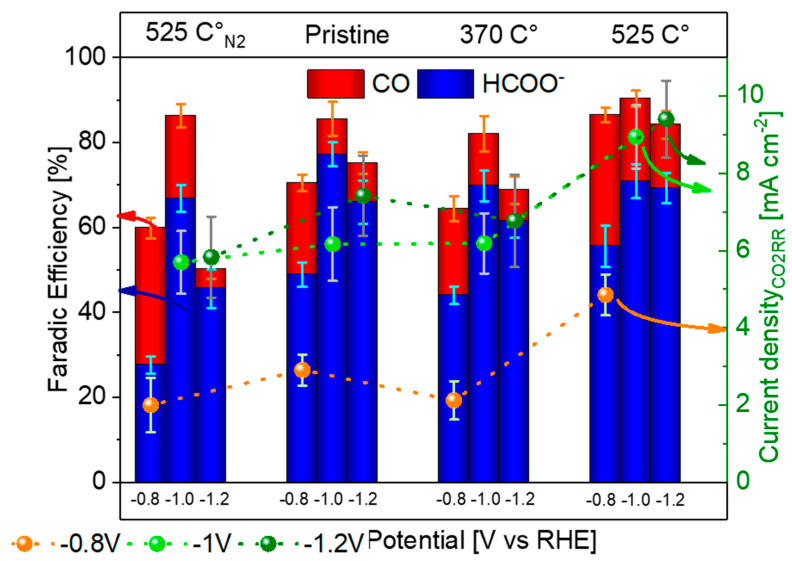
FE towards CO_2_RR products and the current density dedicated to their production. Each sample is labelled by the above legend, and its three studied potential are grouped and identified by the labelled below.

Figure 4 illustrates FE towards CO_2_RR products (HCOO^−^ and CO) and the current density associated with their formation. Each catalyst is labelled at the top of the figure, while the three applied potentials are grouped and labelled along the bottom.

Across all samples, more negative potentials yielded higher current densities and improved CO_2_RR performances, resulting in HER suppression. Above −1 V, the HER increases due to mass transport limitation, a consequence of the H-cell [64]. The as-synthesized catalyst showed an FE of 49% for HCOO^−^ and 22% for CO at −0.8 V. Upon annealing at 370 °C in air, a similar performance was observed, attributed to the incomplete conversion of SnO_x_ to SnO_2_, as evidenced by Raman and XRD analyses. Notably, annealing at 525 °C in air enhanced performance, with an FE of 71% for HCOO^−^ and 19% for CO at −1.0 V. This improvement is attributed to enhanced catalyst oxidation and surface reconstruction, which improved conductivity and the availability of the active sites [65,66]. The HER completes the catalysts FE, and its results are reported in Figure S5A.

In terms of current densities, values at −0.8 V ranged from 2 to 3 mA cm^−2^, except for the 525 °C air-annealed catalyst, which achieved 4.9 mA cm^−2^. At −1.0 V and −1.2 V, all samples displayed similar current densities, ~6 mA cm^−2^ with the 525 °C sample, reaching 9.0 mA cm^−2^. The marginal performances’ increase at −1.2 V are the consequence of the already mentioned cell diffusion limitations.

Interestingly, annealing in an inert atmosphere at 525 °C resulted in lower selectivity and activity, with an FE of 66% for HCOO^−^ and 19% for CO at −1.0 V. The corresponding current densities were also lower, peaking at 5.8 mA cm^−2^, which is lower than the air-annealed counterpart. This difference arises due to fewer oxygen vacancies on the N_2_-annealed catalyst surface, which limits CO_2_ adsorption and activation.

Additionally, the production of formate increased with higher overpotential, at the expensve of the gaseous products. This is consistent with the lower energy barrier for HCOO^−^ formation compared to CO, although CO production is thermodynamically less demanding at lower overpotentials [67,68,69,70]. These results are in line with previous studies [27,65,68,69,71,72]. The maximum selectivity is achieved at −1.0 V. The 525 °C air-annealed catalyst demonstrated superior performance, likely due to the presence of a higher concentration of oxygen vacancies, which are widely recognized for enhancing catalytic activity. Oxygen vacancies increase the number of unsaturated coordination sites, facilitating CO_2_ adsorption and activation while improving electron transfer on the catalyst surface.

Interestingly, Gao et al. reported optimal performance for a catalyst annealed at 300 °C, attributing higher annealing temperatures to a reduction in oxygen vacancies and active sites [68]. However, differences in experimental setups and protocols may explain this apparent discrepancy. Notably, our annealing duration was 2 h, compared to the 30 min process used by Gao et al. Longer annealing times can promote further structural and compositional changes, such as enhanced formation of oxygen vacancies or more extensive reconstruction of the catalyst surface, which likely contribute to the improved performance observed at higher temperatures in our study.

For example, Wei et al. reported that oxygen vacancies in Mn-doped SnO_2_ significantly enhanced CO_2_RR performance by providing more active sites for CO_2_ adsorption during the electrolysis process, leading to improved conversion efficiency [73]. Similarly, Zhao et al. investigated In-doped SnO_2_ and demonstrated that introducing oxygen vacancies not only enhances electrical conductivity but also creates additional active sites, which promotes the catalytic reaction [74]. These improvements are attributed to mechanisms such as narrowing the bandgap and increasing charge carrier density, which collectively enhance the activation of CO_2_ molecules. These findings are consistent with the observations for the air-annealed SnO_2_ catalyst.

This is further supported by recent density functional theory (DFT) calculations, which indicate that oxygen vacancies in SnO_2_ enhance electrocatalytic CO_2_ reduction performance by increasing the number of valence electrons in Sn and lowering the energy barrier of the potential-determining step. This, in turn, facilitates the activation of the C=O bond, enabling selective production of formate and CO [70].

In contrast, the 525 °C N_2_-annealed catalyst exhibited reduced performance due to fewer oxygen vacancies. This is due to the fact that reducing atmospheres during nitrogen annealing, particularly for tin oxides, promotes the reduction of tin to a lower oxidation state [43], as confirmed by the XPS analysis shown in Figure 3. This reduction limits the availability of active sites and, consequently, the catalyst’s ability to efficiently activate CO_2_ molecules.

The CO_2_RR mechanism on tin oxides proceeds via two coupled proton–electron transfer (CPET) steps, with two possible intermediates: *COOH, favoring CO production, and *OCHO, leading to formate formation [68]. Tin oxide exhibits significant selectivity for formate production during the CO_2_ reduction reaction, a property that has been extensively studied. Bagger’s [66] investigation successfully differentiated the catalyst’s selectivity based on the adsorption energy of intermediates compared to that of hydrogen adsorption energy. The interaction between CO_2_ and the catalyst surface is critical, as it defines the formation of intermediates and depends on atomic interactions.

**Figure 5:**
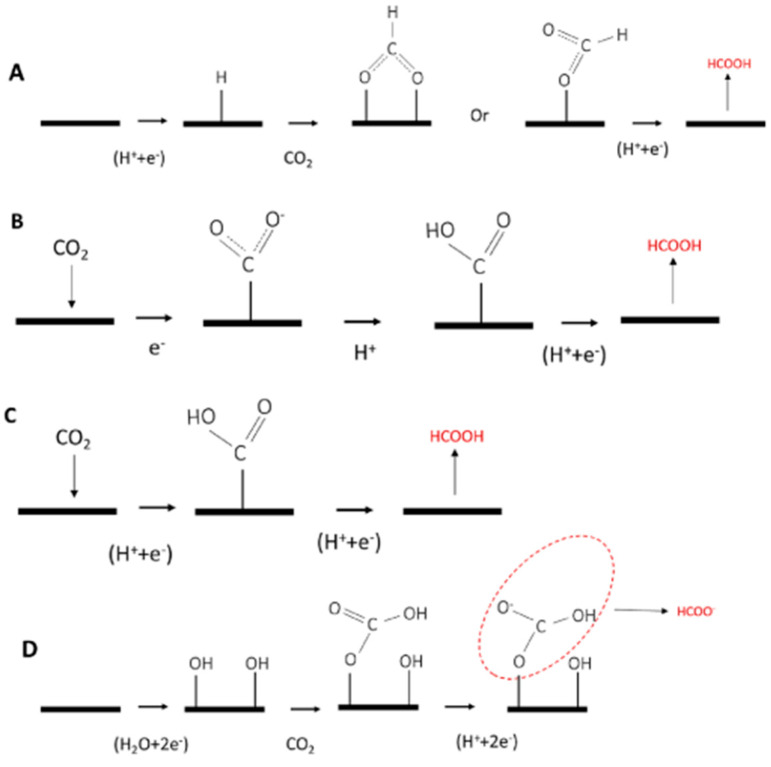
Possible mechanisms for CO_2_ ECR to formate/formic acid. (**A**) monodentate intermediate route; (**B**,**C**) CO_2_^−^ radical intermediate route; and (**D**) surface-bound carbonate intermediate route. Adapted from [75].

If the oxygen atom of the CO_2_ radical is adsorbed onto the surface, it generates the intermediate HCOO*, leading to formate as the final product. Conversely, if the carbon atom is adsorbed, the intermediate *COOH is formed, which is more likely to produce CO but can also result in formate, as illustrated in the schematic plot in Figure 5.

The difference between formic acid and formate arises from the electrolyte pH and reaction path differences. Ewis et al. [75] provided a detailed analysis of these differences, emphasizing the interplay of pH and intermediate stabilization in determining product selectivity. Several studies emphasize the importance of oxygen vacancies in enhancing the catalytic properties of tin oxide. Gao and colleagues highlighted that oxygen vacancies improve O-binding abilities, favoring the formation of HCOO* intermediates. Similar findings have been reported by Ning et al. [29], who extensively investigated the effect of oxygen vacancy concentration on SnO_2_. These studies demonstrate that an increased concentration of vacancies enhances the catalyst’s conductivity by lowering its work function. This, in turn, reduces resistance in the charge transfer process and improves adsorption capacity.

Salvini et al. [70] used ab initio simulations to underline the critical role of oxygen vacancies in the catalytic process and to identify the most active structures for formate production. They reported that the partial reduction of tin dioxide into a thin layer of metallic tin is crucial for the catalytic reaction. The oxidation state of tin, typically Sn^2+^ and Sn^4+^, plays a critical role in catalytic activity.

In operando Raman spectroscopy and DFT were employed to identify the most active species [27,71]. Oxygen vacancies, in particular, lower the energy barrier for the formation of *OCHO intermediates, enhancing selectivity and catalytic activity. The interplay between the oxidation state of tin, oxygen vacancies, and the adsorption energies of reaction intermediates determines the product distribution.

In summary, while all catalysts exhibited similar selectivity toward CO_2_RR, the 525 °C air-annealed sample displayed the highest current density and efficiency. This improvement is directly linked to the formation of oxygen vacancies that enhance CO_2_ adsorption, intermediate stabilization, and electron transfer. The findings highlight the significance of annealing conditions in tuning catalyst properties to achieve optimal performance for electrochemical CO_2_ reduction. This catalyst was selected for further tests in the flow cell to better understand its properties and stability.

### 3.3. Study of the CO_2_RR and Stability of the Air′525 Electrode in a Flow Cell

**Figure 6:**
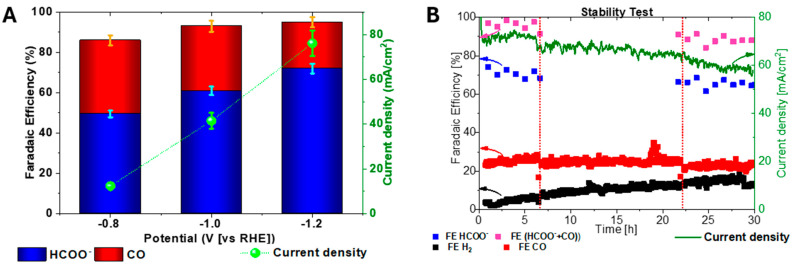
Catalyst Air-525 °C: (**A**): FEs distribution and current densities at selected potentials; (**B**) long-term stability was assessed over 30 h at −1.2 V in 2 M KHCO_3_.

The best-performing catalyst has been tested in a flow cell configuration, using 2 M KHCO_3_ as the electrolyte, to evaluate its performance under optimized conditions. The obtained FE and current densities are shown in Figure 6A. At −0.8 V, the FE for HCOO^−^ reached approximately 48%, while CO contributed around 33%. The current density was 12.4 mA cm^−2^. Increased overpotential improved the current density and the selectivity without suffering of mass transport limitation. At −1.2 V, the FE for HCOO^−^ reached its maximum value of 72%, with CO contributing 23%. The current density increased up to 76.1 mA cm^−2^. The flow cell configuration allows overcoming the mass transport limitations and minimizes the HER, especially due to the more alkaline electrolyte [76]. The FE for H_2_ is reported in Figure S5B.

To assess its stability, the catalyst was tested at −1.2 V, resulting in stable performance during a 30 h test, as shown in Figure 6B. Catalyst stability is as important as its activity and selectivity. As far as we know, the observed stability, selectivity, and current density for the CO_2_RR are comparable to the best results reported in other similar studies at comparable potentials, as shown in Table S1. During the test, the selectivity remained stable, with FE_HCOO^−^_ between 65 and 75% and FE_CO_ at ~25%. The current density started at 82 mA cm^−2^ and gradually decreased to 58 mA cm^−2^. The slight decline in current density is attributed to electrode partial flooding and salt formation, which blocks active sites, hindering CO_2_ access and electron transfer [64,77]. Abrupt current variation at the 7th and 22nd hours were primarily due to the electrolyte replacement during testing, which was necessary to mitigate product accumulation, maintain K^+^ concentration, and restart measurements. The complete FE distribution at the sampling time is reported in Figure S6.

The catalyst exhibited excellent durability during the stability test, maintaining consistent selectivity, as shown in the article. In general, as discussed in the reaction mechanisms, SnO_2_ catalysts operate under dynamic reaction conditions during CO_2_RR, including exposure to highly negative potentials. These conditions can induce structural and compositional changes, such as the partial reduction in SnO_2_ to Sn(II) or metallic Sn [27,65], which can affect catalytic activity and selectivity. Such transformations may decrease faradaic efficiency for CO_2_RR and promote the competing hydrogen evolution reaction. Additionally, oxygen vacancies, known to enhance catalytic activity, could undergo reorganization or loss during extended operations, especially as the catalyst is partially reduced. This reorganization might reduce the availability of active sites for CO_2_ adsorption and activation. Understanding these degradation mechanisms is crucial for optimizing catalyst design. In situ and operando techniques, which are increasingly advancing, offer significant potential for studying not only reaction mechanisms but also long-term degradation under realistic experimental conditions [78,79]. Investigating the interplay between oxygen vacancies, tin oxidation states, and reaction intermediates with such techniques could provide deeper insights into the long-term stability and performance of SnO_2_-based catalysts.

## 4. Conclusions

The present study highlights the influence of annealing conditions and the subsequent modification of structural and electronic properties on SnO_x_ catalysts for CO_2_ reduction to HCOO^−^ and CO. The structural analysis revealed that air annealing at 525 °C optimized the catalyst’s crystallinity and increased oxygen vacancy concentration. This, together with its porous structure morphology, results in an increase in the availability of active sites and facilitates CO_2_ adsorption and activation.

Electrochemical testing demonstrated that the 525 °C air-annealed catalyst achieved superior performance, with an FE of 71% for HCOO^−^ and a current density of 9.0 mA cm^−2^ at −1.0 V, significantly outperforming the N_2_-annealed counterparts. Oxygen vacancies are believed to play a key role in the process by stabilizing *OCHO intermediates, lowering energy barriers, and promoting formate formation, while suppressing HER [66,68,70].

Flow cell experiments further validated the catalyst’s performance, achieving a maximum FE of 72% for HCOO^−^ with a current density of 76.1 mA cm^−2^ at −1.2 V. The stability tests confirmed a consistent performance over 30 h, with a minimal decline attributed to electrolyte replacement. These results underline the critical role of oxygen vacancies and annealing conditions in tuning Sn-based catalysts, offering insights into optimizing catalyst design for an efficient and scalable electrochemical CO_2_ reduction. This study provides a pathway for further advancements in CO_2_RR, demonstrating the potential of SnOx materials for practical applications in renewable energy and sustainable chemical production.

## Figures and Tables

**Table 1:** Crystallite sizes measured by the Rietveld refinement as a function of the annealing temperature and conditions.

Samples	Crystallite Size [nm]	Microstrain (ϵ_μ_)
Pristine	5.1(1)	0.010(2)
Air′370 °C	8.3(1)	0.006(2)
Air′525 °C	9.8(1)	0.004(2)
N_2_′525 °C	7.9(1)	0.006(2)

**Table 2:** XPS relative atomic concentration (at.%) values and O_oxide_/Sn _oxide_ ratio for each sample analyzed. The Oxygen column has been divided into C-O bonds and Oxide bonds.

Samples	Relative Atomic Concentration (at.%)				O_oxide_/Sn
	C_1s_	O_1s_		Sn3d _oxide_	
		C–O Bonds	Oxide		
Pristine	33.2	6.7	40.6	19.5	2.08
Air′370 °C	20.5	3.8	50.5	25.1	2.01
Air′525 °C	19.4	3.5	50.8	26.3	1.93
N_2_′525 °C *	10.4	1.8	58.2	28.0	2.08

* In sample N_2_′525deg, the O/Sn ratio was calculated considering only the % of Sn due to the oxide, while the component, due to the metal (5.3%), was subtracted from the total amount.

## Data Availability

The original contributions presented in this study are included in the article/Supplementary Materials. Further inquiries can be directed to the corresponding author.

## Supplementary Materials

The following supporting information can be downloaded at: https://www.mdpi.com/article/10.3390/nano15020121/s1, Figure S1: Electrochemical setup: (A) H-cell configuration, (Disa Raffaele e F.lli snc) and (B) flow cell. Figure S2: FESEM images of pristine catalyst: (A) top view and (B) cross-section view; Figure S3: Selected Area Electron Diffraction (SAED) patterns of all studied catalysts. The rings in the images were obtained by Circular Hough transform diffraction analysis (A software tool for automated measurement of selected area electron diffraction patterns within Digital Micrograph [41]), and are superimposed on the SAED pattern, showing the position and size of the rings. Figure S4: XPS O1s HR spectra for pristine, annealed in air at 370 °C and annealed in inert atmosphere at 525 °C. Table S1: O 1s XPS peak deconvolution results for the four samples. Table S2: Comparison of Electrocatalytic Performance of Tin-Based Catalysts for CO_2_ Reduction to Formic Acid/formate and CO. Figure S5: FE of H2 of: (A) H-cell experiments and (B) flow cell experiments. Figure S6: Stability test complete FE distribution at sampling time. References [41,73,80,81,82,83,84,85,86,87,88,89,90,91,92] are cited in the supplementary materials.
