# Supplementary material for: Effects of Annealing Conditions on the Catalytic Performance of Anodized Tin Oxide for Electrochemical Carbon Dioxide Reduction
Source: Nanomaterials (Basel). 2025 Jan 16;15(2):121. doi: 10.3390/nano15020121 (PMC11767452; doi:10.3390/nano15020121)
Supplement: Supplementary file 1 [file nanomaterials-15-00121-s001.zip › nanomaterials-3431364-supplementary.pdf]

nanomaterials

Supporting Information

Effects of Annealing Conditions on the Catalytic Performance of Anodized Tin Oxide for Electrochemical Carbon Dioxide Reduction

Nicolò B. D. Monti [1,2], Juqin Zeng [1,2,*], Micaela Castellino [1,2], Samuele Porro [2], Mitra Bagheri [1,2], Candido F. Pirri [1,2], Angelica Chiodoni [1] and Katarzyna Bejtka [1,2,*]

1 Center for Sustainable Future Technologies @POLITO, Istituto Italiano di Tecnologia, Via Livorno 60, 10144 Turin, Italy
2 Department of Applied Science and Technology, Politecnico di Torino, C.so Duca degli Abruzzi 24, 10129 Turin, Italy
* Correspondence: juqin.zeng@polito.it (J.Z.); katarzyna.bejtka@polito.it (K.B.)

Academic Editor: George Z. Kyzas

Received: 31 December 2024
Revised: 11 January 2025
Accepted: 13 January 2025
Published: 16 January 2025

Citation: Monti, N.B.D.; Zeng, J.; Castellino, M.; Porro, S.; Bagheri, M.; Pirri, C.F.; Chiodoni, A.; Bejtka, K. Effects of Annealing Conditions on the Catalytic Performance of Anodized Tin Oxide for Electrochemical Carbon Dioxide Reduction. *Nanomaterials* **2025**, *15*, 121. https://doi.org/10.3390/nano15020121

Copyright: © 2025 by the authors. Licensee MDPI, Basel, Switzerland. This article is an open access article distributed under the terms and conditions of the Creative Commons Attribution (CC BY) license (https://creativecommons.org/licenses/by/4.0/).

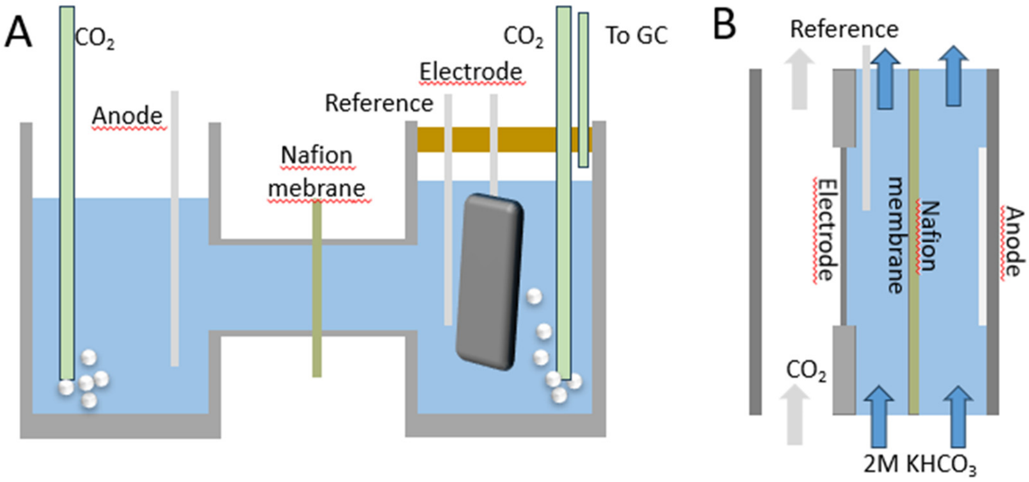

Figure S1. Electrochemical set up: A) H-cell configuration, (Disa Raffaele e F.lli snc) and B) flow cell.

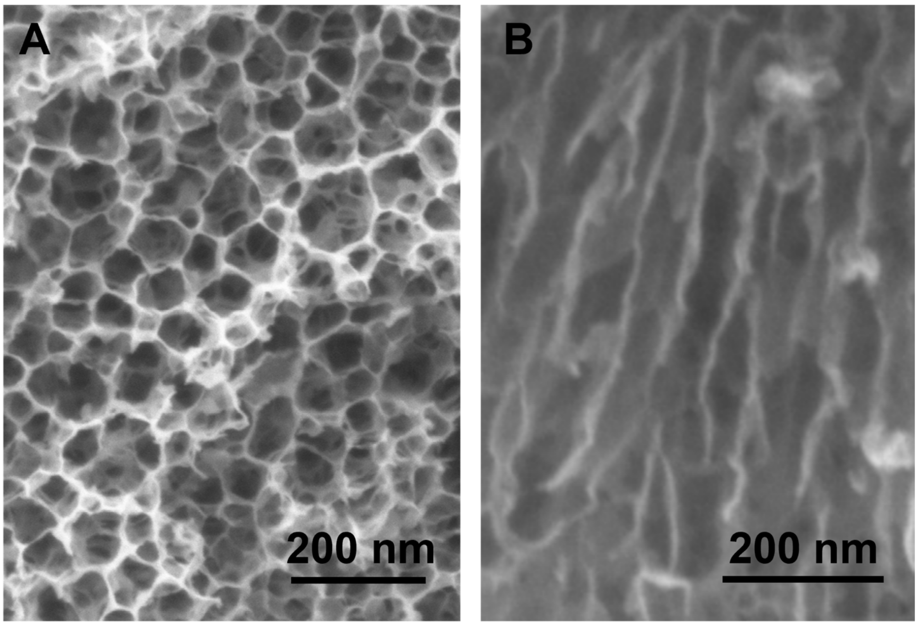

Figure S2. FESEM images of pristine catalyst: (A) top view and (B) cross-section view.

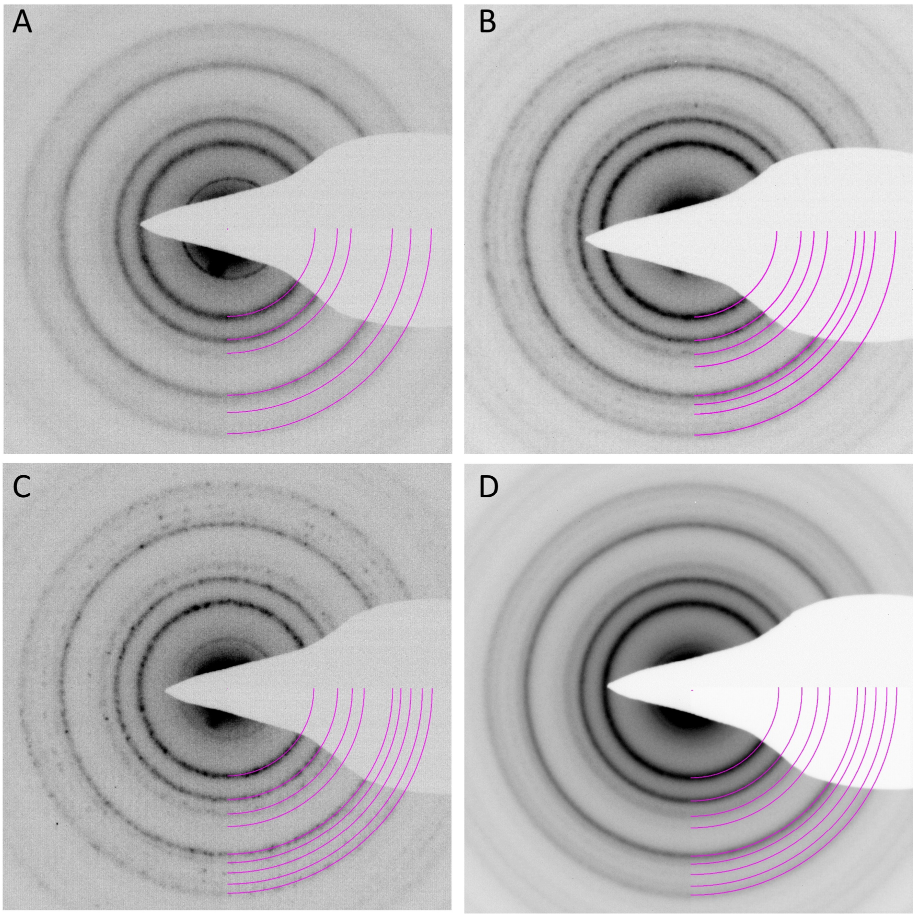

Figure S3. Selected Area Electron Diffraction (SAED) patterns of all studied catalysts: (A) Pristine, (B)Air′-370deg, (C)Air′-525deg and (D)N2′-525deg. The rings in the images were obtained by Circular Hough transform diffraction analysis (A software tool for automated measurement of selected area electron diffraction patterns within Digital Micrograph [1]), and are superimposed on the SAED pattern, showing the position and size of the rings. These are polycrystalline SnO_2 (Tin Oxide, JCPDS 00-041-1445).

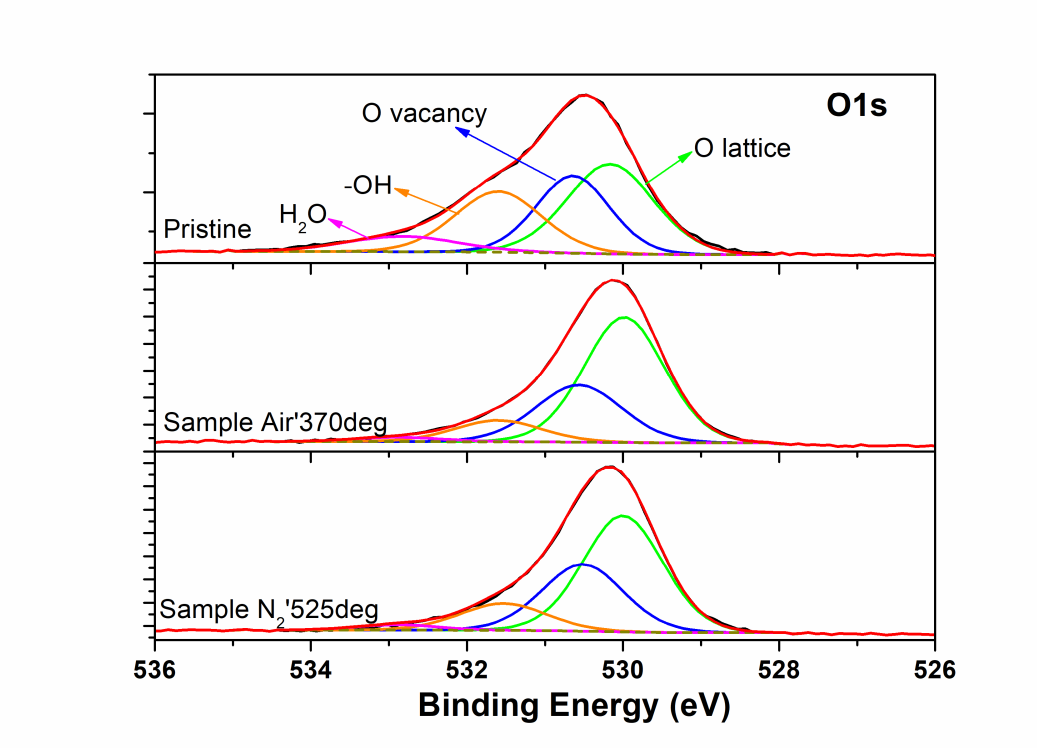

Figure S4. XPS O1s HR spectra for pristine, annealed in air at 370 °C and annealed in inert atmosphere at 525 °C.

Table S1. O 1s XPS peak deconvolution results for the four samples.

Samples	O1s XPS peak deconvolution results			
	Lattice O	O_{vac}	-OH	H_2O
Pristine	38.1	27.8	25.7	8.4
Air′370°C	58.5	28.5	10.7	2.3
Air′525°C	53.1	31.2	13.2	2.5
N_2′525°C	54.9	27.4	14.3	3.4

Table S2. Comparison of Electrocatalytic Performance of Tin-Based Catalysts for CO_2 Reduction to Formic Acid / formate and CO.

Electrocatalysts	Electrolyte	FE_{CO2RR} [%]	Applied potential [V vs. RHE]	Current density [mA cm^{-2}]	Stability [h]	Ref
Mesoporous SnO_x Nanoparticles	2 M $KHCO_3$	95	-1.2	72	30	This work
Wavy SnO_2	0.5 M KHCO3	91	−1.0	22 S	18	[2]
Mesoporous SnO_2 nanosheets	0.5 M KHCO3	90	−1.3	8.3	12	[3]
Porous SnO_2/CC	0.5 M NaHCO3	95	−1.6 (V vs. Ag/AgCl)	45	24	[4]
Porous SnO2 nanosheets	0.5 M KHCO3	92.4	−0.7	NA	10	[5]
Ultra-small SnO_2 NPs	1 M KHCO3	80	−1.21	145	NA	[6]
Ultrathin SnO_2 QWs	0.1 M KHCO3	87.3	−1.156	13.7	7	[7]
Pt atom/SnO_2	0.1 M KHCO3	82.1 ± 1.4	−1.2	12.9	8	[8]
In-SnO_2 NWs	0.5 M KHCO3	85	−1.04	6.02	12	[9]
Mn-doped SnO_2	0.1 M KHCO3	91.6	−1.03	21.2	8	[10]
VO-rich N-SnO_2 NS	0.1 M KHCO3	>90	−0.9	6.7	10	[11]
SnO_2@N-CNW	0.5 M NaHCO3	90	−0.8	25	20	[12]
NC-SnO_2@CC	0.5 M KHCO3	93	−0.7	44.3	24	[13]
SnO_2/Sn	0.5 M KHCO3	93 ± 1	−1.0	28.7	9	[14]
SnO_2/Py-CNTO	0.1 M KHCO3	96	−1.29	27.5	32	[15]

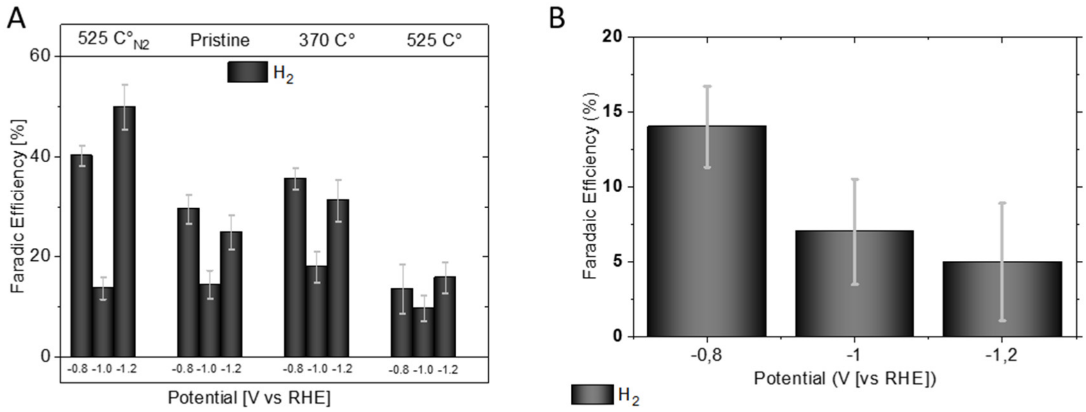

Figure S5. FE of H_2 of: A) H-cell experiments and B) flow cell experiments.

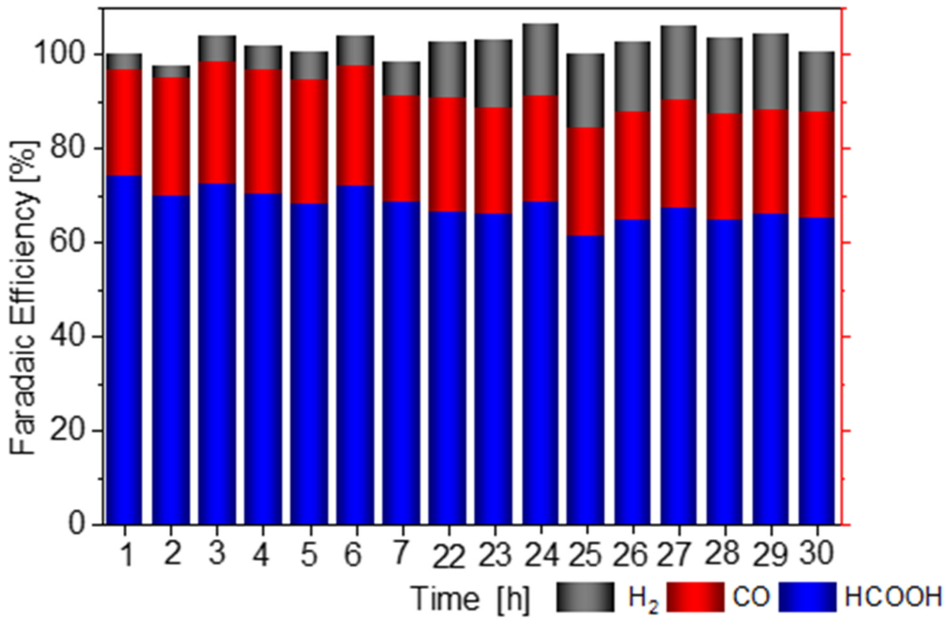

Figure S6. Stability test complete FE distribution at sampling time.

REFERENCES

1. D.R.G. Mitchell, Circular Hough transform diffraction analysis: A software tool for automated measurement of selected area electron diffraction patterns within Digital MicrographTM, Ultramicroscopy 108 (2008) 367–374. https://doi.org/10.1016/j.ultramic.2007.06.003.

2. Z. Chen, T. Fan, Y.Q. Zhang, J. Xiao, M. Gao, N. Duan, J. Zhang, J. Li, Q. Liu, X. Yi, J.L. Luo, Wavy SnO2 catalyzed simultaneous reinforcement of carbon dioxide adsorption and activation towards electrochemical conversion of CO2 to HCOOH, Appl Catal B 261 (2020). https://doi.org/10.1016/j.apcatb.2019.118243.
3. F. Wei, T. Wang, X. Jiang, Y. Ai, A. Cui, J. Cui, J. Fu, J. Cheng, L. Lei, Y. Hou, S. Liu, Controllably Engineering Mesoporous Surface and Dimensionality of SnO2 toward High-Performance CO2 Electroreduction, Adv Funct Mater 30 (2020). https://doi.org/10.1002/adfm.202002092.
4. F. Li, L. Chen, G.P. Knowles, D.R. MacFarlane, J. Zhang, Hierarchical Mesoporous SnO 2 Nanosheets on Carbon Cloth: A Robust and Flexible Electrocatalyst for CO 2 Reduction with High Efficiency and Selectivity , Angewandte Chemie 129 (2017) 520–524. https://doi.org/10.1002/ange.201608279.
5. G. Liu, Z. Li, J. Shi, K. Sun, Y. Ji, Z. Wang, Y. Qiu, Y. Liu, Z. Wang, P.A. Hu, Black reduced porous SnO2 nanosheets for CO2 electroreduction with high formate selectivity and low overpotential, Appl Catal B 260 (2020). https://doi.org/10.1016/j.apcatb.2019.118134.
6. C. Liang, B. Kim, S. Yang, Y.L. Yang Liu, C. Francisco Woellner, Z. Li, R. Vajtai, W. Yang, J. Wu, P.J.A. Kenis, P.M. Ajayan, High efficiency electrochemical reduction of CO 2 beyond the two-electron transfer pathway on grain boundary rich ultra-small SnO 2 nanoparticles, J Mater Chem A Mater 6 (2018) 10313–10319. https://doi.org/10.1039/C8TA01367E.
7. S. Liu, J. Xiao, X.F. Lu, J. Wang, X. Wang, X.W. (David) Lou, Efficient Electrochemical Reduction of CO 2 to HCOOH over Sub-2 nm SnO 2 Quantum Wires with Exposed Grain Boundaries, Angewandte Chemie International Edition 58 (2019) 8499–8503. https://doi.org/10.1002/anie.201903613.
8. X. Zhou, E. Song, Z. Kuang, Z. Gao, H. Zhao, J. Liu, S. Sun, C.-Y. Mou, H. Chen, Tuning selectivity of electrochemical reduction reaction of CO2 by atomically dispersed Pt into SnO2 nanoparticles, Chemical Engineering Journal 430 (2022) 133035. https://doi.org/10.1016/j.cej.2021.133035.
9. D. Tan, W. Lee, K.T. Park, Y.E. Jeon, J. Hong, Y.N. Ko, Y.E. Kim, Promoting CO2 reduction to formate selectivity on indium-doped tin oxide nanowires, Appl Surf Sci 613 (2023) 155944. https://doi.org/10.1016/j.apsusc.2022.155944.
10. Y. Wei, J. Liu, F. Cheng, J. Chen, Mn-doped atomic SnO 2 layers for highly efficient CO 2 electrochemical reduction, J Mater Chem A Mater 7 (2019) 19651–19656. https://doi.org/10.1039/C9TA06817A.
11. Z. Li, A. Cao, Q. Zheng, Y. Fu, T. Wang, K.T. Arul, J. Chen, B. Yang, N.M. Adli, L. Lei, C. Dong, J. Xiao, G. Wu, Y. Hou, Elucidation of the Synergistic Effect of Dopants and Vacancies on Promoted Selectivity for CO 2 Electroreduction to Formate, Advanced Materials 33 (2021). https://doi.org/10.1002/adma.202005113.
12. B. Zhang, L. Sun, Y. Wang, S. Chen, J. Zhang, Well-dispersed SnO2 nanocrystals on N-doped carbon nanowires as efficient electrocatalysts for carbon dioxide reduction, Journal of Energy Chemistry 41 (2020) 7–14. https://doi.org/10.1016/j.jechem.2019.04.022.
13. B. Zhang, S. Chen, B. Wulan, J. Zhang, Surface modification of SnO2 nanosheets via ultrathin N-doped carbon layers for improving CO2 electrocatalytic reduction, Chemical Engineering Journal 421 (2021) 130003. https://doi.org/10.1016/j.cej.2021.130003.

14. S. Ning, J. Wang, D. Xiang, S. Huang, W. Chen, S. Chen, X. Kang, Electrochemical reduction of SnO_2 to Sn from the Bottom: In-Situ formation of SnO_2/Sn heterostructure for highly efficient electrochemical reduction of carbon dioxide to formate, J Catal 399 (2021) 67–74. https://doi.org/10.1016/j.jcat.2021.04.028.
15. Y. Zhang, H. Xu, D. Niu, X. Zhang, Y. Zhang, Pyridine Grafted on SnO_2 -Loaded Carbon Nanotubes Acting as Cocatalyst for Highly Efficient Electroreduction of CO_2, ChemSusChem 14 (2021) 2769–2779. https://doi.org/10.1002/cssc.202100541.
